# SPOCK1 and POSTN are valuable prognostic biomarkers and correlate with tumor immune infiltrates in colorectal cancer

**DOI:** 10.1186/s12876-022-02621-2

**Published:** 2023-01-07

**Authors:** Caiqin Gan, Mengting Li, Yuanyuan Lu, Ganjing Peng, Wenjie Li, Haizhou Wang, Yanan Peng, Qian Hu, Wanhui Wei, Fan Wang, Lan Liu, Qiu Zhao

**Affiliations:** 1 Department of Gastroenterology, Zhongnan Hospital of Wuhan University, Wuhan, 430000 China; 2 Hubei Clinical Center and Key Lab of Intestinal and Colorectal Diseases, Wuhan, 430000 China

**Keywords:** SPOCK1, POSTN, Cancer-associated fibroblasts, Tumor microenvironment, Tumor-infiltrating immune cells, Colorectal cancer

## Abstract

**Background:**

Immune cells and stromal cells in the tumor microenvironment play a vital role in the progression of colorectal cancer (CRC). The study aimed to screen valuable prognostic biomarkers in CRC based on stromal and immune scores.

**Method:**

The ESTIMATE algorithm was used to calculate the immune and stromal scores of CRC samples in TCGA. Then samples were divided into high and low score groups based on the median value of the scores. Differentially expressed genes (DEGs) associated with immune and stromal scores were screened. WGCNA and univariate COX regression analysis were performed to further identify key prognostic genes. Analysis of scRNA-seq for CRC was used for verifying the main source of the key genes. The prognostic value of they was validated based on The Gene Expression Profiling Interactive Analysis and GSE17536 dataset. TIMER and CIBERSORT algorithms were applied to analyze the correlations among key genes and tumor-infiltrating immune cells. Several pairs of colon cancer tissue were used to be proven.

**Result:**

1314 upregulated and 4 downregulated genes were identified, which were significantly enriched in immune-related biological processes and pathways. Among these DEGs, SPOCK1 and POSTN were identified as key prognostic genes and mainly expressed in cancer-associated fibroblasts for CRC. High expression of SPCOK1 and POSTN was associated with advanced clinical stage, T stage, N stage, and poor prognosis of CRC. The results from CIBERSORT and TIMER revealed that SPOCK1 and POSTN were associated with tumor-infiltrating immune cells, especially macrophages and neutrophils. Meanwhile, in several pairs of human colorectal tissue samples, SPOK1 and POSTN were found to be significantly overexpressed in colorectal tissue compared with para-cancer tissue, and macrophage surface markers CD68 (co-expressed by M1 and M2 macrophages) and CD206 (M2-specific macrophage expression) were also overexpressed in cancer tissue. Besides, SPOCK1 and POSTN expression were positively correlated with the expression of immune checkpoints.

**Conclusion:**

Collectively, our results indicate that SPOCK1 and POSTN associated with CAF may be novel prognostic biomarkers in CRC and correlate with immune infiltrates.

**Supplementary Information:**

The online version contains supplementary material available at 10.1186/s12876-022-02621-2.

## Background

Colorectal cancer (CRC) ranks as the third most common malignancy around the world with high incidence and high mortality [1]. The current treatments for CRC mainly include surgery, chemotherapy, radiation therapy, and other targeted therapies. However, the prognosis of CRC has never been satisfying, especially for patients with advanced stage [2]. Genetic and epigenetic changes are closely related to the occurrence of tumors and the prognosis of CRC [3, 4]. Therefore, understanding the molecular mechanism of CRC is essential for developing preventive and treatment strategies of CRC.

Accumulating evidence confirmed the importance of the tumor microenvironment (TME) in cancer development. The complex interaction between tumor cells and TME contributes to tumor progression and impacts the patient’s clinical outcome [5–7]. In this context, tumor-infiltrating immune cells and stromal cells, which are the main components of TME, have received extensive attention. Previous studies have demonstrated that stromal cells promote tumor angiogenesis and extracellular matrix remodeling during tumor progression [8]. Meanwhile, numerous studies revealed that immune cells in the TME play important roles in tumorigenesis and are attractive therapeutic targets[9–11]. Furthermore, it has been reported that infiltrating immune cells are involved in metastasis [12]. In these biological processes, immune and stromal genes may influence the prognosis of cancer patients by regulating the abundance and function of immune cells and stromal cells. However, there is a lack of effective biomarkers related to immune and stromal scores that can predict CRC patients’ prognosis. Therefore, identifying these biomarkers is conducive to cancer diagnosis and explore new molecular targeted therapies.

With the emergence of high-throughput detection technology and the development of bioinformatics, transcriptome analysis has been widely used to assess the abundance of different types of cells in the TME. In this study, we applied the ESTIMATE scoring method to calculate the immune score and stromal score of each CRC sample from The Cancer Genome Atlas (TCGA) database. SPOCK1 and POSTN were identified as prognostic key genes associated with immune score and stromal score. The relationships between their expression with the clinical characteristics and prognosis of colorectal cancer were validated by kinds of analyses. CIBERSORT algorithm and Tumor Immune Estimation Resource (TIMER) were used to elucidate the association of SPOCK1 and POSTN expression with immune cell infiltration level. The high expression levels of SPOK1 and POSTN, as well as the positive expressions of CD68 and CD206 in several pairs of human CRC specimens, were detected by immunohistochemistry and immunofluorescence.

In conclusion, our finding indicated that high expression of SPOCK1 and POSTN predicted poor prognosis in CRC. Meanwhile, SPOCK1 and POSTN were enriched in a variety of immune-related pathways and were related to the regulation of tumor immune cells and the expression of multiple immune checkpoint genes. In addition, SPOK1 and POSTN were significantly higher expressed in cancer tissues compared to adjacent colon cancer tissues, as well as CD68(co-expressed by M1 and M2 macrophages) and CD206 (M2-specific macrophage expression), suggesting that they might be valuable prognostic biomarkers and targets for immunotherapy.

## Materials and methods

### Specimen collection

A total of 8 pairs of primary tumor tissues and corresponding normal tissues were collected from CRC patients who received surgical treatment at Zhongnan Hospital of Wuhan University (Wuhan, China). All patients were diagnosed by original histopathological detection and none of them received preoperative adjuvant chemotherapy or radiotherapy. The patients with non-curative resection, cancer recurrence, severe injury of vital organs, or a history of autoimmune diseases were excluded. The detailed patient characteristics are displayed in Additional file 4: Table S1. Samples of the collected tissues were preserved in liquid nitrogen. The collection of clinical specimens was approved by the clinical research institution review committee and ethics review committee of the Zhongnan Hospital (Number: 2020110), verbal informed consent was obtained from the patients for their anonymized information to be published in this article.

### Data collection and processing

RNA-seq data in FPKM format and corresponding clinical information of CRC have been downloaded from The Cancer Genome Atlas (TCGA) database (https://portal.gdc.cancer.gov/). There were 556 CRC samples with gene expression data in the TCGA. All samples were included in gene expression analysis. After excluding 45 patients with incomplete information, a total of 511 patients with complete clinical information such as survival time, survival status, age, gender, stage, T/M/N stage were included in survival analysis and clinical correlation analysis. The detailed patient characteristics of TCGA are displayed in Additional file 5: Table S2. Moreover, as a validation dataset, the normalized gene expression profile of GSE17536 was downloaded from Gene Expression Omnibus (GEO) database (https://www.ncbi.nlm.nih.gov/geo/). This dataset containing 177 CRC samples with complete clinical information.

### Differentially expressed genes (DEGs) screening

The immune and stromal scores of each CRC sample were calculated based on the ESTIMATE algorithm, which was performed by R package “estimate” [13]. The samples were divided into high-score and low-score groups respectively according to the median of the immune and stromal scores. Differentiation analysis of the gene expression was performed by using the R package “limma”, and DEGs were screened from the comparison between the high-score group and the low-score group. |log2 fold change (FC) | ≥ 1.0 and false discovery rate (FDR) < 0.05 were set as the threshold for filtering DEGs. The intersection of the up-regulated and down-regulated DEGs in the immune and stromal score groups was identified by the online Venn diagrams tool (Venny 2.1, https://bioinfogp.cnb.csic.es/tools/venny/).

### Functional enrichment analysis of the DEGs

Gene Ontology (GO) and Kyoto encyclopedia of Genes and Genomes (KEGG) analyses ( www.kegg.jp/kegg/kegg1.html ) were performed for intersecting DEGs through “clusterProfiler”, “enrichplot”, “ggplot2” in R software. The terms with* p* value < 0.05 were considered statistically significant.

### Construction of weighted co-expression network and identification of clinical significant modules

Weighted gene co-expression network analysis (WGCNA) was performed to identify hub gene modules associated with clinical. The expression profile data and phenotype data matrix of 1318 DEGs were selected for WGCNA [14]. R package “WGCNA” was performed, and the scale-free topology fit index for several powers and mean connectivity were calculated to assess the soft-thresholding power for the network construction. The soft threshold value was determined when the scale independence value was 0.9. The adjacency was turned into a topological overlap matrix (TOM), which could measure the network connectivity of genes. Based on the TOM dissimilarity with a minimum gene group size of 30 for the gene dendrogram, hierarchical clustering was carried out to classified similar genes into modules. The merge cut height was set as 0.25 to merge some modules founded on the dissimilarity of module eigengenes.

We calculated the correlation between co-expression modules and clinical features to identify clinically relevant modules. For intramodular analysis, Module membership (MM) and gene significance (GS) were calculated. Here we selected the significant module with a threshold of* p* value < 0.05.

### Key genes screening and validation

Genes with higher MM and GS in the selected module would be defined as hub genes, and the ranked top 30 were selected. Second, prognostic genes were obtained from DEGs of the intersection by univariate Cox regression analysis with *p* < 0.01 as the cutoff value. Third, Venn analysis was conducted to select the intersection of genes in the above two steps, and the genes were identified as key prognostic genes for subsequent analysis. Survival analyses (overall survival (OS) and disease-free survival (DFS)) of key genes were carried out based on TCGA and the Genotype-Tissue Expression (GTEx) data in the Gene Expression Profiling Interactive Analysis (GEPIA) database (http://gepia.cancer-pku.cn/). For further validation, Kaplan-Meier curves based on GSE17536 were generated using the R package “survival”. *p* < 0.05 was considered significant.

### ScRNA-seq data preparation and processing

Single-cell transcriptome files of GSE110009 and GSE120065 were downloaded from the GEO database These two single-cell transcriptome profiles were integrated for downstream analysis. For nFeature_RNA < 50, mitochondrial sequencing count > 5% of the cells were excluded. The batch effect in the study removed with regularized negative binomial regression by “Seurat” package. The t-distributed stochastic neighbor embedding (t-SNE) analysis was performed for dimension reduction analysis. We used the “Seurat” package to find cluster biomarkers and clustered the cells. Ultimately, the results indicate that SPOCK1 and POSTN are mainly expressed in CAF for CRC (Additional file 1: Fig.S1A–D).

### Gene set enrichment analysis (GSEA)

According to the median expression value of key genes, CRC patients in TCGA were divided into high and low expression groups. Gene Set Enrichment Analyses (GSEA) were performed to identify enriched KEGG pathways in the two groups using the JAVA program (https://www.broadinstitute.org/gsea). The number of permutations was set at 1000 for each analysis. Nominal *p* < 0.05, and a false discovery rate (FDR) *q* < 0.25 were considered statistically significant. Multiple GSEA plots were visualized by “plyr”, “ggplot2”, and “grid” packages in R.

### Correlation analysis between key genes and immune microenvironment

The CIBERSORT[15] (https://cibersort.stanford.edu/) algorithm was performed to evaluate the relative abundances of 22 immune cells in all CRC samples based on RNA-seq from the TCGA database. Samples with *p* < 0.05 were considered qualified for further analysis. The 22 immune cells between high and low expression groups of key genes were visualized by using the “vioplot” package. The Tumor Immune Estimation Resource (TIMER) (https://cistrome.shinyapps.io/timer/) online database is also known as a web server for analyzing tumor-infiltrating immune cells[16]. We downloaded the immune infiltration levels of TCGA-CRC patients. The relationship between key gene expression and six immune cell subtypes (B cells, CD4+ T cells, CD8+ T cells, dendritic cells, macrophages, and neutrophils) was determined.

### 10 immunohistochemical staining and tissue immunofluorescence staining

Colon cancer specimens were fixed in 4% paraformaldehyde solution for 3 days and treated with paraffin embedding technology. After dewaxed, rehydrated, and antigenic recovery, the primary antibody added before blocking with BSA was treated with EDTA antigenic repair buffer (pH8.0). The dilution ratio of primary antibody was anti-SPOCK1(1:200 dilution, ab229935, Abcam), anti-POSTN (1:200, bs-4994R, Bioss), anti-cd68(1:100, SC-20,060, Santa), anti-206(1:100,18704-AP, Proteintech). Immunohistochemistry was performed with a DAB staining kit (GeneTech Company, Ltd., Shanghai, China) for the protein expression of SPOCK1, POSTN, CD68, CD206, PD1, and TIM-3 in human colon cancer samples. For tissue immunofluorescence, the flat slices were incubated at 4 C overnight. The next day, the secondary antibody covered with the corresponding species of the primary antibody was added and incubated for 1 h at room temperature. The secondary antibody was counterstained with a fluorescent secondary antibody Cy3 conjugated goat anti-rabbit and FITC conjugated goat anti-mouse IgG (H + L) (1:50 dilution) and 4, 6-diamidino-2-phenylindole (DAPI) and sealed with an anti-fluorescence quenching capsule. All of the images were captured by a Nikon NIS Elements BR light microscope (Nikon, Tokyo, Japan). Analyzing the fluorescence intensity of each sample in different regions with the ImageJ software to determine the average staining intensity.

### Statistical analysis

Statistical analyses were performed using R version 3.6.3 and SPSS V25.0. The Kaplan–Meier method was conducted to plot survival curves, and a log-rank was used as the statistical significance test. Differences among variables were compared using t-tests, nonparametric tests. The correlation of gene expression was evaluated by Spearman’s correlation. If not specified above, *p* < 0.05 was considered statistically significant.

## Results

### Analysis of differential gene expression profiles of immune and stromal scores in CRC

**Fig. 1:**
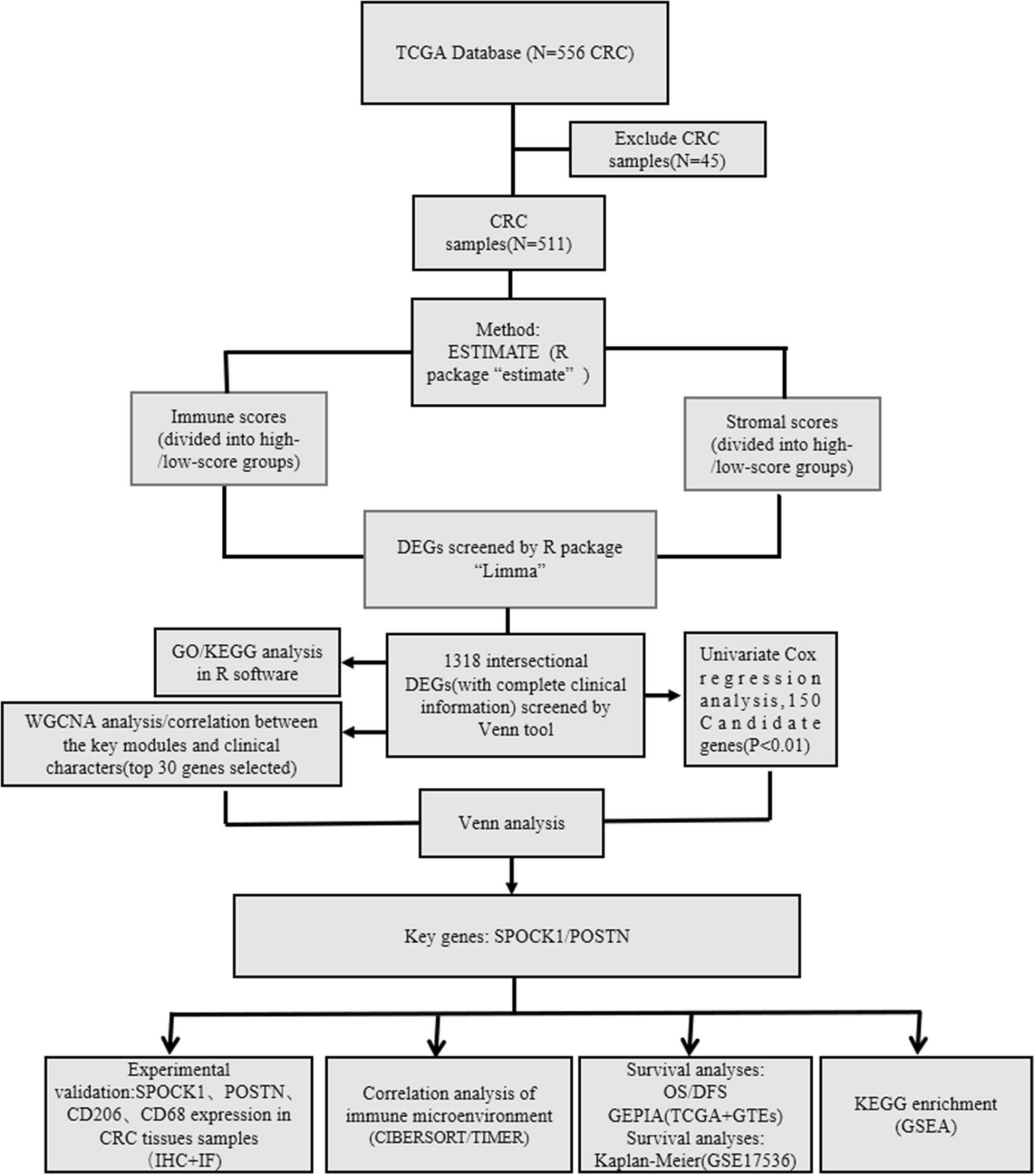
The flowchart of the procedure for the study

**Fig. 2:**
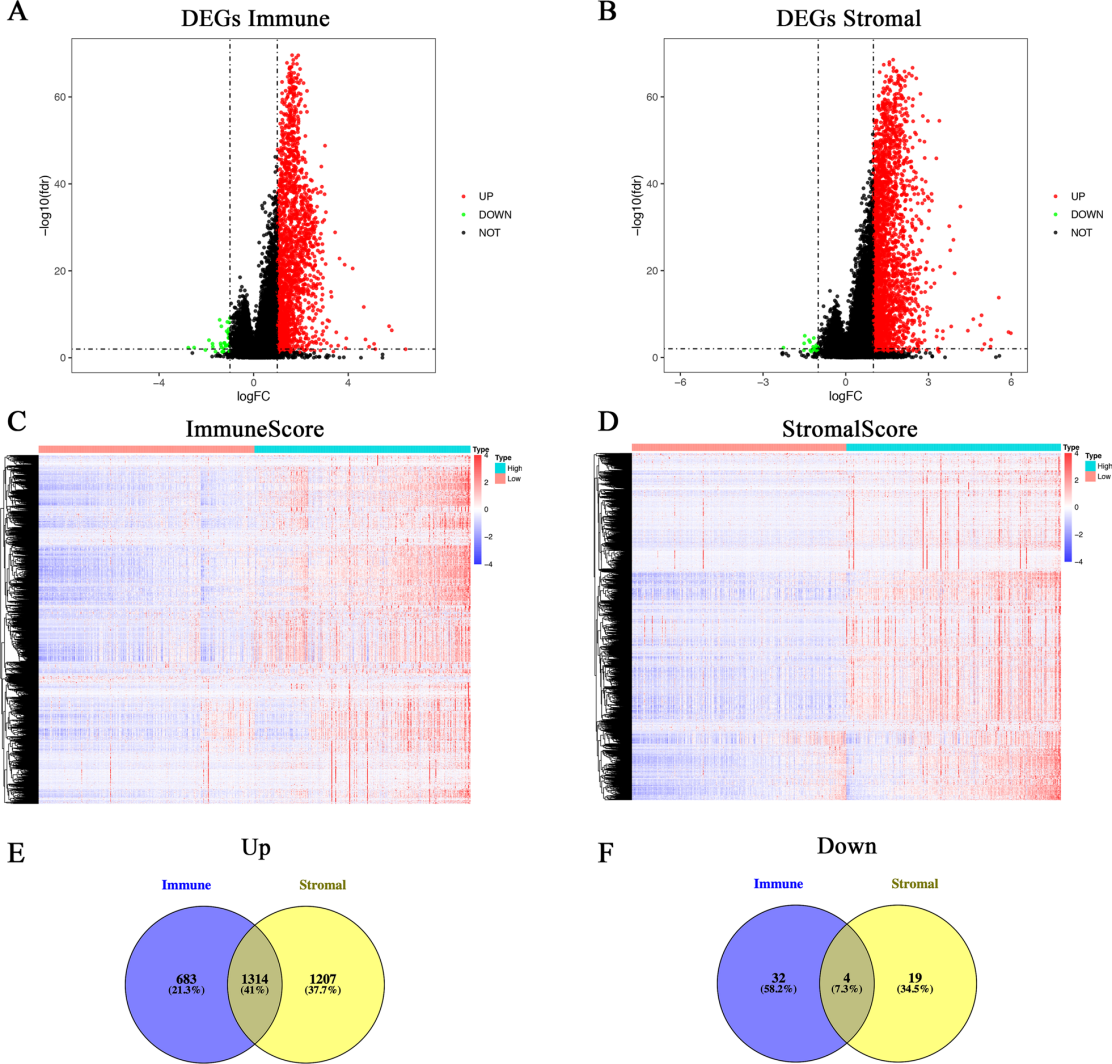
Identification of DEGs based on immune and stromal scores in CRC.** A**,** C** Volcano plot and heatmap for DEGs generated by comparison of the high immune score group versus the low immune score group (*p* < 0.05, fold change > 1). **B**, **D** Volcano Plot and Heatmap for DEGs generated by comparison of the high stromal score group versus the low stromal score group (*p* < 0.05, fold change > 1). **E** Venn diagram showing common up-regulated DEGs in the high immune/stromal score groups. **F** Venn diagram showing common down-regulated DEGs in the low immune/stromal score groups. DEGs are differentially expressed genes

The procedure for the study is shown in the flowchart in detail (Fig. 1). The immune scores and stromal scores of all CRC samples were calculated using the ESTIMATE algorithm and stratified into high and low groups according to their median levels. The gene expression profiles of the high- and low-score groups were compared. A total of 2033 DEGs were obtained based on immune scores (high vs. low), consisting of 1997 up-regulated and 36 down-regulated (Fig. 2 A, C, E, F). Similarly, 2521up-regulated and 23 down-regulated genes were identified from stromal scores (high vs. low) (Fig. 2B, D, E, F). The Venn plots showed that 1314 up-regulated genes overlapped in the high immune/stromal score groups and 4 down-regulated genes overlapped in the low immune/stromal score groups.

### Functional enrichment analyses of DEGs in the intersection

**Fig. 3:**
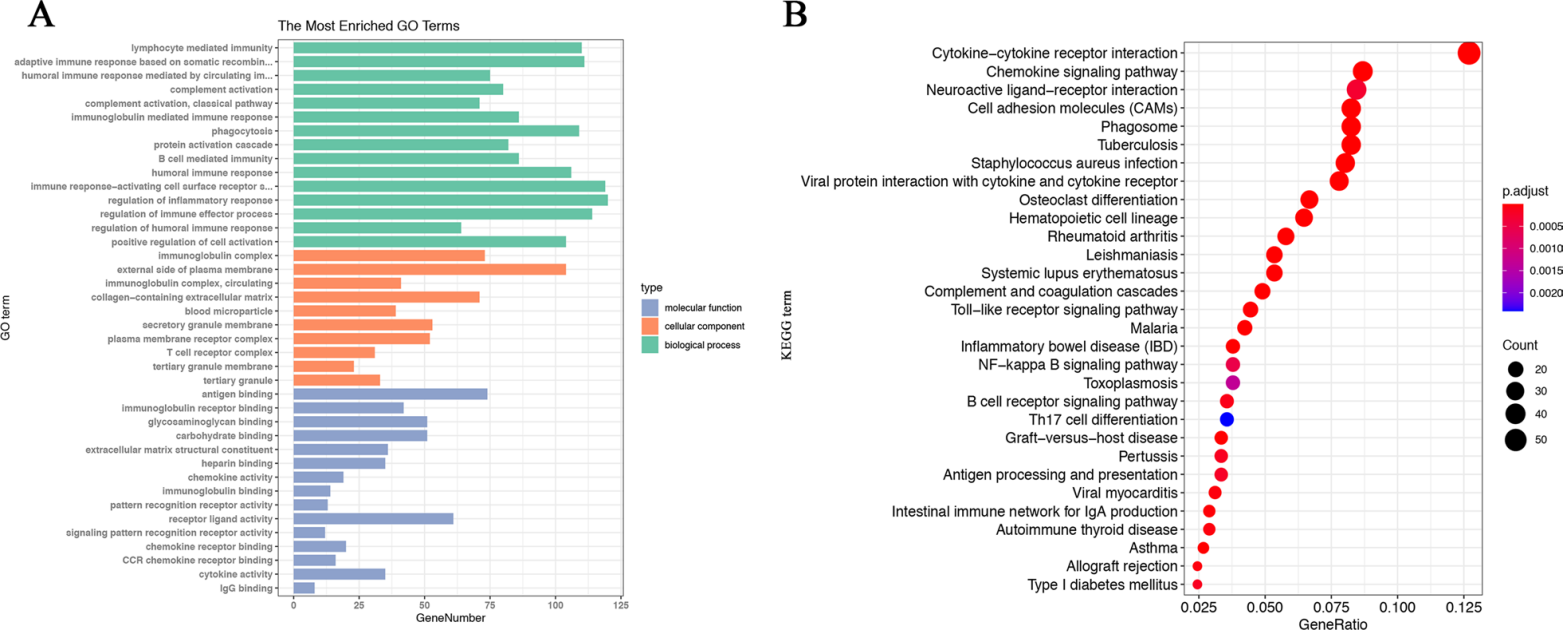
Functional enrichment analyses of DEGs in the intersection. **A** GO enrichment analysis for 1318 DEGs in the intersection. **B** KEGG enrichment analysis for 1318 DEGs in the intersection. GO, gene ontology; KEGG, Kyoto Encyclopedia of Genes and Genomes

Gene Ontology (GO) and Kyoto Encyclopedia of Genes and Genomes (KEGG) enrichment analyses were performed to evaluate the biological functions and pathways involved in these DEGs in the intersection. The results of GO enrichment analysis showed that these DEGs were almost enriched in immune-related terms. lymphocyte-mediated immunity, immunoglobulin complex, and antigen-binding were the most significant enrichment terms among biological processes, cellular components, and molecular functions (Fig. 3 A). KEGG analysis also displayed that these DEGs were mainly enriched in cytokine-cytokine receptor interaction, chemokine signaling pathway, and neuroactive ligand-receptor interaction (Fig. 3B). The enrichment results suggested that these DEGs participate in immune-related activities and might regulate TME in CRC.

### Construction of co-expression network and identification of hub genes

To further narrow candidate genes and screen hub genes related to clinicopathological factors, a total of 1318 DEGs in the intersection were included in the co-expression network analysis. 511 CRC patients with complete information including survival, stage, and T/M/N stage were selected.

**Fig. 4:**
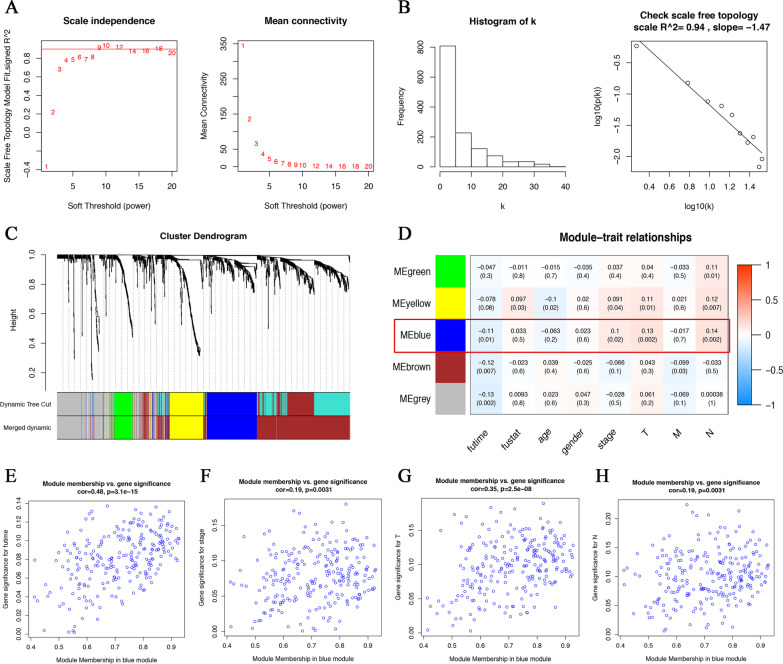
WGCNA analysis and the correlation between the key module and the clinical characters of CRC. **A** Scale independence and mean connectivity analysis. **B** Visualized scale-free topology. **C** The gene cluster dendrogram was obtained by hierarchical clustering of adjacency-based dissimilarity. **D** The relationships between clustered modules and clinical traits. **E**–**H** The Module-trait membership plot shows that the blue module is significantly correlated with survival time, clinical stage, T stage, and N stage. WGCNA, weighted gene co-expression network analysis

The soft threshold was set to 9, with the lowest mean connectivity (Fig. 4 A). As shown in Fig. 4B, the logarithm of the node with connectivity k log(k) was negatively correlated with the logarithm of the node probability log(p(k)), and the correlation coefficient reached 0.94. The MEDissThres was set as 0.25 to merge similar modules, and five modules were eventually generated (Fig. 4 C). Among them, there were 240 genes in the blue module, 509 genes in the brown module, 85 genes in the green module, 321 genes in the grey module, and 163 genes in the yellow module. A grey module is a group of genes that could not fit into other modules. Correlation analysis indicated that the blue module was significantly associated with the clinical features of CRC (Fig. 4D). Furthermore, the module membership in the blue module also has a significant correlation with clinical features such as survival time, clinic stage, T stage, and N stage (Fig. 4E, H). We calculated the connectivity between genes in the blue module, and the top 30 genes with the highest connectivity were screened as hub genes for subsequent analysis.

### Selection and validation of key genes associated with survival

**Fig. 5:**
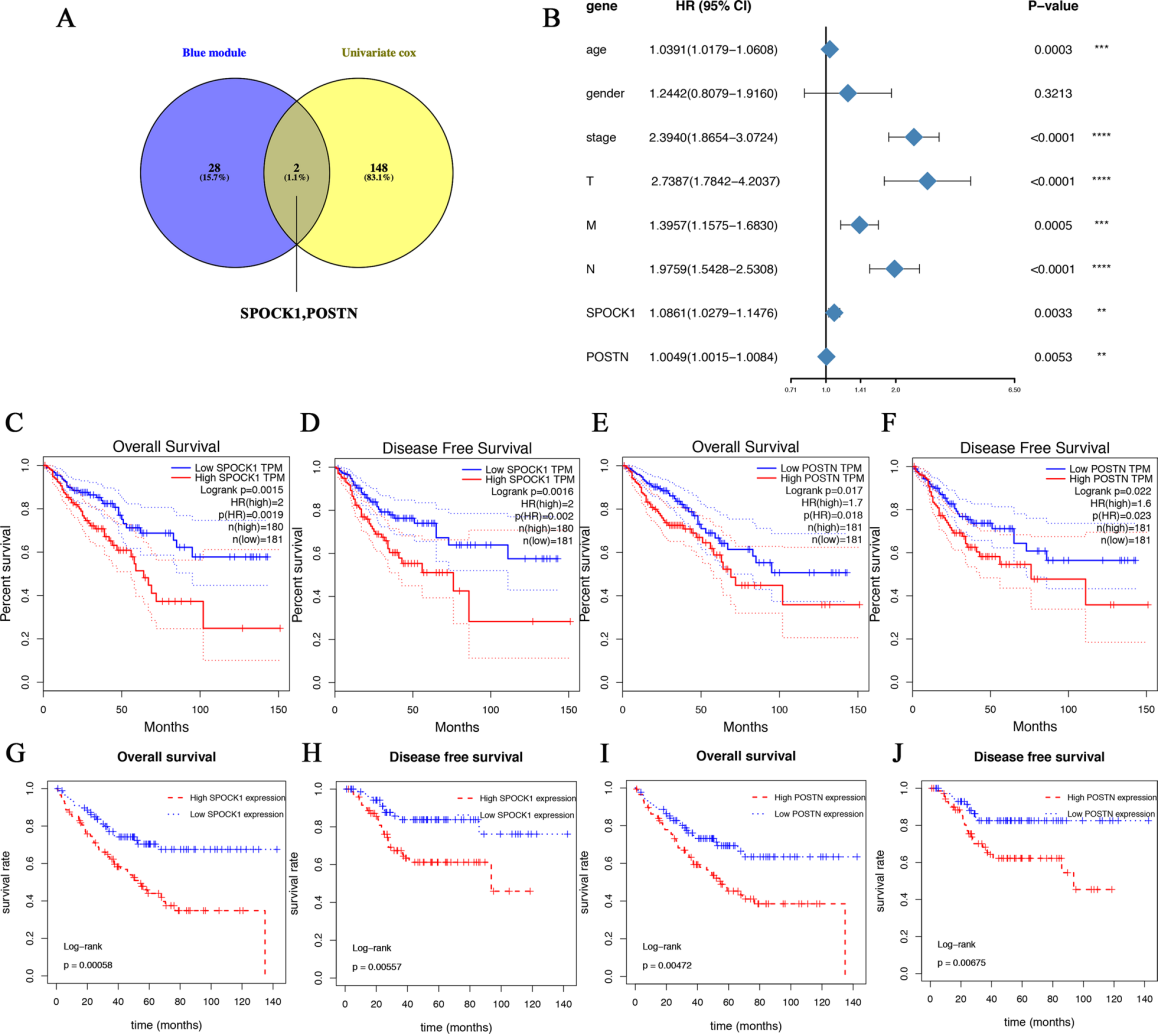
Identification and validation of key prognostic genes. **A** Venn plots show the intersection between hub genes in the blue module and top significant genes in univariate COX. **B** Forest map showing the hazard ratio of SPOCK1, POSTN, and clinical characters in CRC. **C**, **D** OS and DFS of SPOCK1 high and low expression CRC patients were analyzed in GEPIA with TCGA data. **E**, **F** OS and DFS of POSTN high and low expression CRC patients were analyzed in GEPIA with TCGA data. **G**, H OS and DFS of SPOCK1 high and low expression CRC patients were analyzed in the GSE17536 dataset. **I**, **J** OS and DFS of POSTN high and low expression CRC patients were analyzed in the GSE17536 dataset. GEPIA, the gene expression profiling interactive analysis; OS, overall survival; DFS, disease-free survival

Univariate Cox regression analysis was used to evaluate the prognostic values of DEGs in the intersection. Of the 1318 DEGs, 150 were identified to be significantly associated with the prognosis of CRC (*p* < 0.01). Venn analysis was used to obtain the intersection of 30 hub genes in the co-expression analysis and significant genes in univariate COX analysis (Fig. 5 A). As a result, SPOCK1 and POSTN were identified as key genes associated with survival. As shown in the forest plot, SPOCK1 (Hazard Ratio = 1.0861, 95% CI of ratio: 1.0279–1.1476, *p* = 0.0033), POSTN (Hazard Ratio = 1.0049, 95% CI of ratio: 1.0015–1.0084, *p* = 0.0053), age (Hazard Ratio = 1.0391, 95% CI of ratio: 1.0179–1.0608, *p* = 0.0003), stage (Hazard Ratio = 2.3940, 95% CI of ratio: 1.8654–3.0724, *p* < 0.0001), T stage (Hazard Ratio = 2.7387, 95%C I of ratio: 1.7842–4.2037, *p* < 0.0001), M stage (Hazard Ratio = 1.3957, 95% CI of ratio: 1.1575–1.6830, *p* = 0.0005), N stage (Hazard Ratio = 1.9759, 95% CI of ratio: 1.5428–2.5308, *p* < 0.0001) were relevant to OS among patients with CRC (Fig. 5B), while the results of multivariate COX regression analysis for OS among patients with CRC showed no statistical significance (Additional file 2: Fig. S2) .

The survival analysis of GEPIA data revealed that CRC patients with high expression of SPOCK1 (Hazard Ratio = 2, Logrank *p* = 0.0015, Fig. 5 C) and POSTN (Hazard Ratio = 1.7, Logrank *p* = 0.017, Fig. 5E) had poor OS. Meanwhile, high expression of SPOCK1 (Hazard Ratio = 2, Logrank *p* = 0.0016, Fig. 5D) and POSTN (Hazard Ratio = 1.6, Logrank *p* = 0.022, Fig. 5 F) were also significantly correlated with DFS. To validate the prognostic value of hub genes, we performed survival analyses based on an independent subset GSE17536. Consistent with the previous results, high expressions of SPOCK1 and POSTN were significantly associated with worse OS (*p* < 0.01, Fig. 5G and I) and DFS (*p* < 0.01, Fig. 5 H and 5 J). In the summary above, it may not be independent prognostic factors for CRC, however, SPOCK1 and POSTN are still associated with the prognosis of CRC as important risk factors.

### Association of SPOCK1 and POSTN expression with clinicopathological factors

**Fig. 6:**
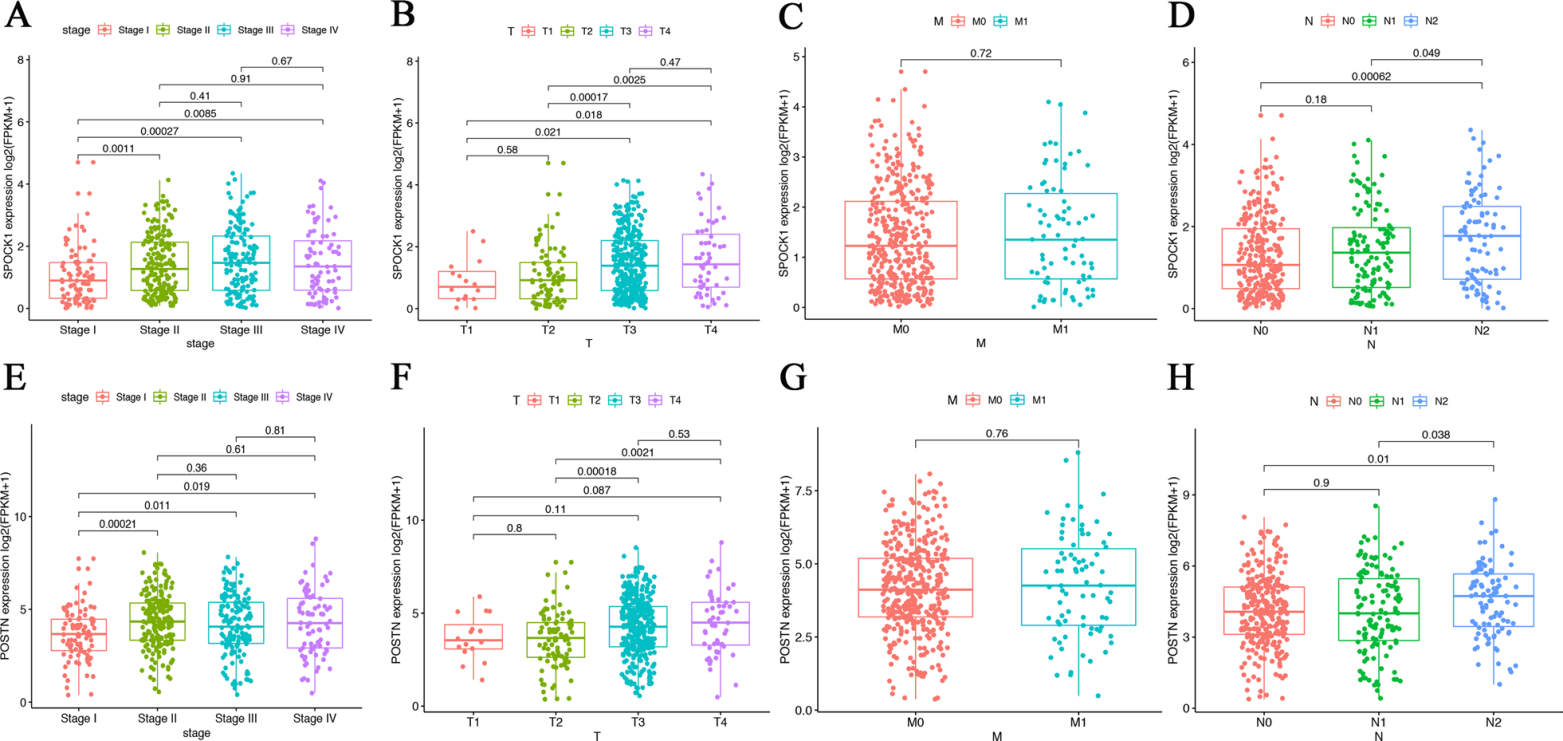
The association between SPOCK1 and POSTN expression with clinical features of CRC. **A**–**D** The correlation between with clinical stage, T stage, N stage, and M stage in low expression and high expression of SPOCK1 groups. **E**–**H** The correlation between clinic stage, T stage, N stage, and M stage in low expression and high expression of POSTN groups

To investigate the relationships between key prognostic genes and the clinical characteristics of CRC patients in TCGA, we compared the expression levels of SPOCK1 and POSTN in different clinical stages, T stage, M stage, and N stage. The results showed that differential expression of SPOCK1 was significantly associated with the clinical stage (Fig. 6 A), T stage (Fig. 6B), and N stage (Fig. 6 C). Moreover, differential expression of POSTN was also related to stage (Fig. 6E), T stage (Fig. 6 F), and N stage (Fig. 6G). However, the expression of SPOCK1 and POSTN had no significant influence on the M stage (Fig. 6D H).

### Gene set enrichment analysis of key genes

**Fig. 7:**
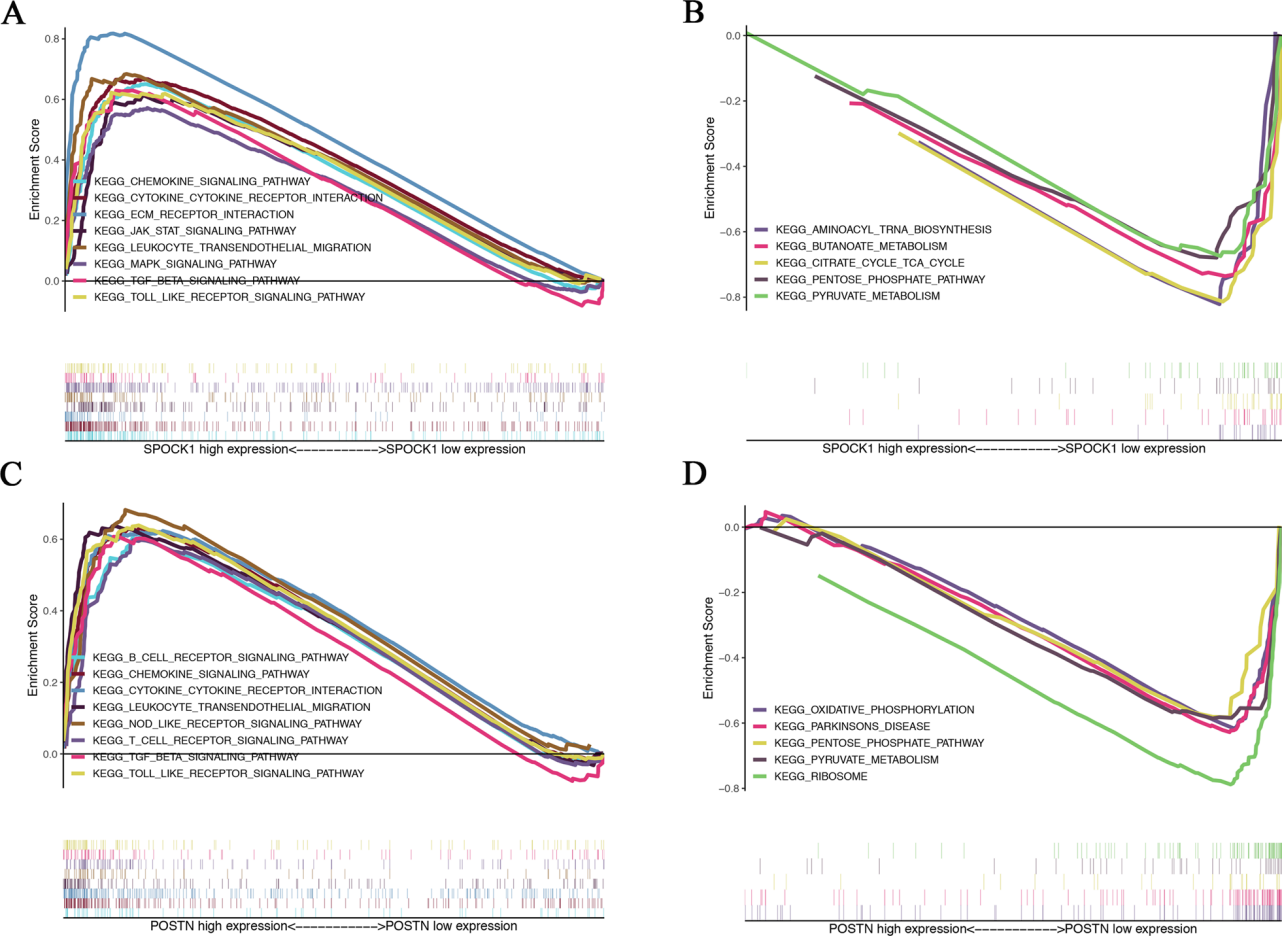
KEGG pathway enrichment analysis of SPOCK1 and POSTN. **A** The enriched gene sets in the SPOCK1 high expression group by GSEA. **B** The enriched gene sets in the SPOCK1 low expression group by GSEA. **C** The enriched gene sets in the POSTN high expression group by GSEA. D The enriched gene sets in the POSTN low expression group by GSEA. Only gene sets with NOM *p* < 0.05 and FDR q < 0.25 were considered significant. GSEA, gene set enrichment analysis

To further explore the underlying mechanism, we performed GSEA between high-expression groups and low-expression groups based on the median levels of SPOCK1 and POSTN expression, respectively. As shown in Fig. 7, the genes in SPOCK1 high-expression group were mainly enriched in immune-related pathways like chemokine signaling, cytokine-cytokine receptor interaction, and ECM-receptor interaction (Fig. 7 A). However, the genes in the SPOCK1 low expression group were more significantly enriched in metabolic-related pathways, such as aminoacyl tRNA biosynthesis, butanoate metabolism, and citrate cycle TCA cycle (Fig. 7B). In the POSTN high expression group, genes were also mainly enriched in the B cell receptor signaling pathway, chemokine signaling pathway, cytokine-cytokine receptor interaction, and other immune-related pathways (Fig. 7 C). While, the genes in POSTN low-expression group were enriched significantly in oxidative phosphorylation, Parkinsons disease, pentose phosphate pathway (Fig. 7D). These results suggested that SPOCK1 and POSTN might have potential regulatory effects on the immune microenvironment of CRC.

### Correlation of SPOCK1 and POSTN expression with immune infiltration levels

**Fig. 8:**
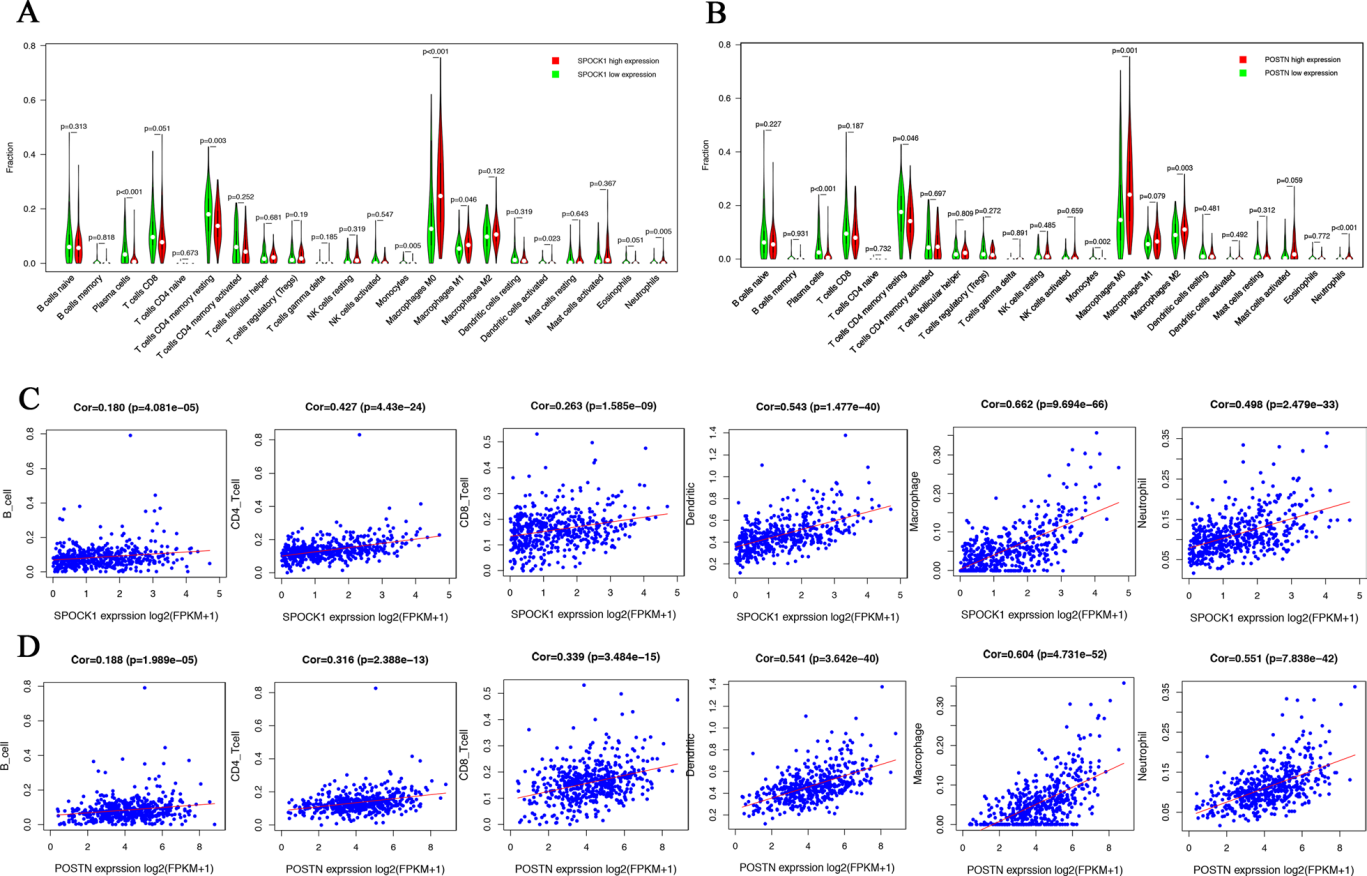
Correlation of infiltration immune cells with SPOCK1 and POTSN expression in CRC. **A** The violin plot shows the differences of 22 CIBERSORT immune cells between CRC samples with low or high SPOCK1 expression. **B** The violin plot shows the differences of 22 CIBERSORT immune cells between CRC samples with low or high POSTN expression. C The scatter plot shows the correlation of SPOCK1 expression with 6 kinds of immune cells in TIMER. D The scatter plot shows the correlation of POSTN expression with 6 kinds of immune cells in TIMER

To confirm the relationships between SPOCK1 and POSTN expression with the immune microenvironment. We calculated the relative abundances of the 22 immune cells in all CRC samples using the CIBERSORT algorithm. The violin diagram provided visualization of the differences in 22 immune cells between the high and low expression groups of SPOCK1 and POSTN. The results showed that plasma cells (*p* < 0.001), resting CD4 memory T cells (*p* = 0.003), monocytes (*p* = 0.005), and activated dendritic cells (*p* = 0.023) were in higher proportions in the SPOCK1 low expression group, whereas macrophages M0 (*p* < 0.001), macrophages M1 (*p* = 0.046), and neutrophils (*p* = 0.005) were in higher proportions in the SPOCK1 high expression group (Fig. 8 A). The POSTN low expression group also had a higher proportion of plasma cells (*p* < 0.001), resting CD4 memory CD4 T cells (*p* = 0.046), and monocytes (*p* = 0.002), whereas the POSTN high expression group had a higher proportion of macrophages M0 (*p* = 0.001), macrophages M2 (*p* = 0.003), and neutrophils (*p* < 0.001) (Fig. 8B). Additionally, we used the TIMER algorithm to determine the correlations between SPOCK1 and POSTN expression with 6 kinds of infiltration immune cells. The analysis showed that SPOCK1 and POSTN expression had significantly positive correlations with infiltrating levels of B cells, CD4+ T cells, CD8+ T cells, dendritic cells, macrophages, and neutrophils (Fig. 8 C, D). From the above results, both SPOCK1 and POSTN were related to immune cell infiltration in CRC, especially macrophages and neutrophils.

### SPOCK1, POSTN, CD68, and CD206 expressions in clinical samples of CRC and para-cancer

**Fig. 9:**
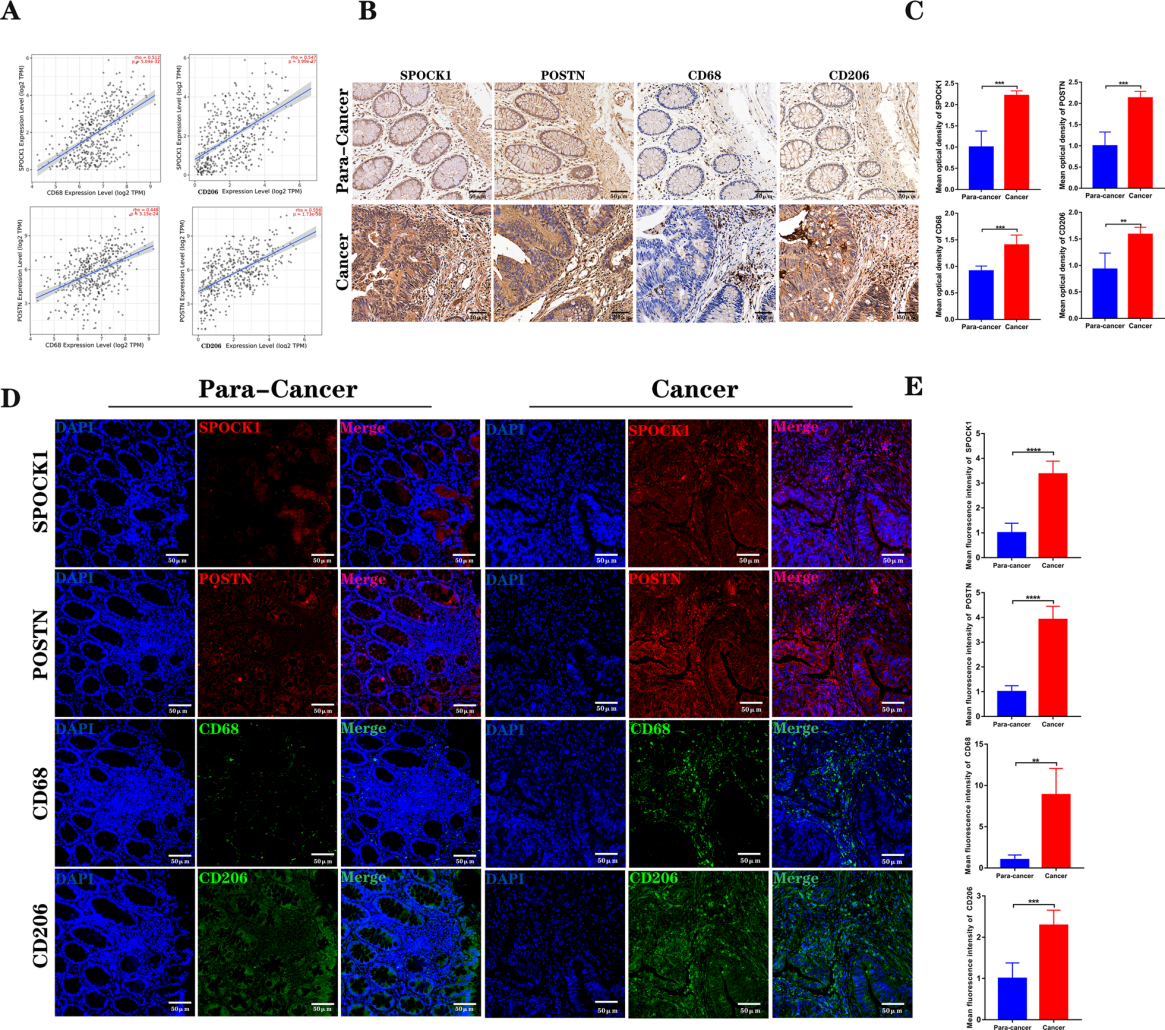
The CAF-associated SPOCK1, POSTN, CD68, and CD206 expression in clinical samples of Colon cancer and Para-Colon cancer. **A** Positive correlation of CD68 and CD206 with SPOCK1and POSTN expression in human colon cancer tissues downloaded from TIMER. **B** Representative tissue IHC results for SPOCK1, POSTN, CD68, and CD206. Scale bar = 50 μm. **C** Mean optical density for SPOCK1, POSTN, CD68, and CD206, respectively. **D** Representative tissue immunofluorescence images for SPOCK1, POSTN, CD68, and CD206. Scale bar = 50 μm. E Mean fluorescence intensity for SPOCK1, POSTN, CD68, and CD206, respectively. *p < 0.05; **p < 0.01; ***p < 0.001

Previous studies reported that SPOCK1 and POSTN expressions related to cancer-associated fibroblasts (CAF) played an important role in tumors, and our results suggested that SPOCK1 and POSTN associated with poor prognosis mainly express in CAF for CRC[17–19]. To verify whether CAF-associated SPOCK1 and POSTN expressions are associated with the immune cell infiltration of CRC, especially with macrophages, we detected the expression of SPOCK1, POSTN, CD68, and CD206, which are related to CAF in 8 cases of colon cancer and corresponding para-cancerous tissues by immunohistochemistry and immunofluorescence assay, and found that compared with para-cancerous tissues, SPOCK1 and POSTN were significantly up-regulated in cancer tissues (Fig. 9B–E), while CD68 and CD206 were also significantly overexpressed in cancer tissues(Fig. 9B–E). The human colon cancer tissues data downloaded from TIMER also showed that SPOCK1 and POSTN were positively correlated with CD68 and CD206 in colon cancer tissues (Fig. 9 A). Above results, it is not difficult to find that SPOCK1 and POSTN related to CAF are co-expressed with CD68 and CD206 in colon cancer, which indicates that the CAF-associated POCK1 and POSTN expressions have an important influence on macrophages, especially M2 macrophages.

### Correlation of SPOCK1 and POSTN expression with immune checkpoint genes expression

**Fig. 10:**
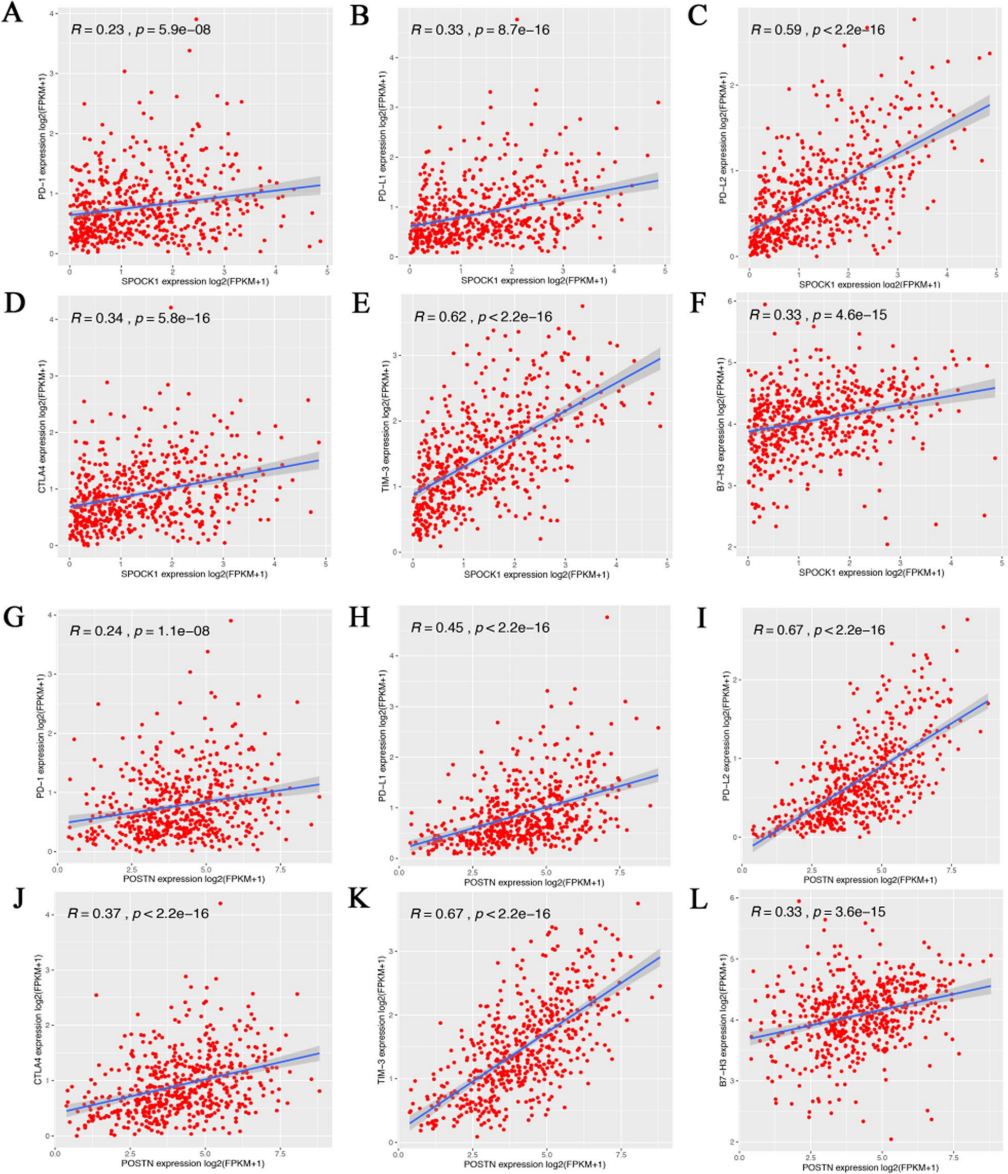
Correlation of immune checkpoints with SPOCK1 and POSTN expression in CRC. **A**–**F** SPOCK1 expression is positively correlated with the expression of PD-1, PD-L1, PD-L2, CTLA4, TIM-3, and B7-H3. **G**–**I** POSTN expression is positively correlated with the expression of PD-1, PD-L1, PD-L2, CTLA4, TIM-3, and B7-H3 in TCGA.

Immune checkpoint molecules have been identified to suppress antitumor immune responses in solid tumors. Therefore, we analyzed the correlation between SPOCK1 and POSTN expression with immune checkpoints gene expression such as PD-L1, PD-1, PD-L2, CTLA4, TIM-3, and B7-H3. The results showed that SPOCK1 expression was significantly positively correlated with PD-1 (R = 0.23, *p* = 5.9e–08, Fig. 10 A), PD-L1 (R = 0.33, *p* = 8.7e–16, Fig. 10B), PD-L2 (R = 0.59, *p* < 2.2e–16, Fig. 10 C), CTLA4 (R = 0.34, *p* = 5.8e–16, Fig. 10D), TIM-3 (R = 0.62, *p* < 2.2e–16, Fig. 10E), and B7-H3 (R = 0.33, *p* = 4.6e–15, Fig. 10 F). Similarly, POSTN was also positively correlated with PD-1 (R = 0.24, *p* = 1.1e–08, Fig. 10G), PD-L1 (R = 0.45, *p* < 2.2e–16, Fig. 10 H), PD-L2 (R = 0.67, *p* < 2.2e–16, Fig. 10I), CTLA4 (R = 0.37, *p* < 2.2e–16, Fig. 10 J), TIM-3 (R = 0.67, *p* < 2.2e–16, Fig. 10 K), and B7-H3 (R = 0.33, *p* = 3.6e–15, Fig. 10 L). In addition, our immunohistochemical staining results of CRC tissues also showed that the expressions of SPOCK1 and POSTN were highly positively correlated with the expressions of immune checkpoints PD-1 and TIM-3 (Additional file 3: Fig. S3). These suggested that high expression of SPOCK1 and POSTN might be correlated with tumor immune evasion in CRC.

## Discussion

In the presented study, we screened out differentially expressed genes (DEGs) based on the immune and stromal scores of CRC samples in TCGA. CAF-related SPOCK1 and POSTN were identified as key prognostic genes for CRC. In the validation phase, SPOCK1 and POSTN have highly expressed in tumor cells and CAF for CRC, and the expressions associated with CAF have a poor prognosis and late clinical stage of CRC. In addition, we found that SPOCK1 and POSTN related to CAF are involved in immune activity, co-expressed with M2-type macrophages, and are associated with immune cell infiltration and immune escape.

In TME, immune and stromal cells play a critical role in tumorigenesis. Growing studies have shown that the composition of the TME can predict patient prognosis and serve as an important target for cancer therapy [20–23]. It has been reported that the immune and stroma classification of CRC is relevant to precision immunotherapy [24]. In recent years, immunotherapy has made great progress in the treatment of CRC, especially in patients with microsatellite instability [25, 26]. Because the molecular mechanism of the TME in CRC is still unclear, and there is a lack of effective immune biomarkers, current immunotherapy has only achieved significant clinical effects in a subset of CRC patients [27]. Therefore, it is necessary to take an in-depth understanding of the composition of TME and to investigate novel prognostic biomarkers for the immunotherapy of CRC.

Here, we use the ESTIMATE method to calculate the immune and stromal scores of CRC samples from TCGA. The CRC samples were divided into two groups based on the median value of the immune scores and stromal scores. DEGs between the two groups were selected. 1314 upregulated DEGs were in common among the high immune/stromal score groups and 4 downregulated DEGs were in common among the low immune/stromal score groups. Functional enrichment analysis showed that the biological processes and main pathways of these DEGs were related to immune-related terms, such as lymphocyte-mediated immunity and cytokine-cytokine receptor interaction. These results indicate that these DEGs are highly correlated with immune response and tumor immune microenvironment. Based on the obtained 1318 DEGs, the WGCNA method was used to screen clinically relevant hub genes; while univariate Cox regression analysis was used to identify survival-related genes. The intersection involved SPOCK1 and POSTN as the key prognostic genes, and scRNA-seq analysis for CRC showed that SPOCK1 and POSTN were mainly expressed in CAF. Studies reported that SPOCK1 and POSTN expressions related to CAF were associated with tumorous prognosis. We validated that high expression of SPOCK1 and POSTN was associated with the poor prognosis of CRC patients by applying OS and DFS analyses based on GEPIA and GSE17536. This is consistent with previous studies on POSTN [28, 29], but the prognostic value of SPOCK1 in CRC has not been reported before. In addition, our tissue specimens also showed significant overexpression of SPOCK1 and POSTN in tumor cells and CAF for colon cancer. Besides, high expression of SPOCK1 and POSTN was correlated to higher clinical stage, T stage, and N stage, but not with M stage, which is consistent with the WGCNA analysis. Accordingly, the results indicated the potential of SPOCK1 and POSTN as prognostic markers and therapeutic targets for TME in CRC.

SPOCK1 encodes a Ca2^+^-binding matricellular glycoprotein, which belongs to the SPARC family. SPOCK1 has been demonstrated to function in cell proliferation, migration, and apoptosis in certain types of cancer, such as pancreatic ductal adenocarcinoma [30], lung cancer [31], colorectal cancer[32], and hepatocellular carcinoma [33], indicating that SPOCK1 plays an important role in oncogenesis. To elucidate the biological roles of SPOCK1, we conducted GSEA to explore the relevant pathways. It was shown that the SPOCK1 high expression group is mainly enriched in immune-related pathways, such as chemokine signaling pathway, cytokine-cytokine receptor interaction, JAK-STAT signaling pathway. In addition, tumorigenesis-related pathways, such as ECM receptor interaction and MAPK signaling pathway, were also enriched in the SPOCK1 high expression group. Previous studies have shown that SPOCK1 is a key regulator of ECM and can mediate EMT in cancer cells [34]. Altogether, these results suggested that SPOCK1 promotes tumor progression by affecting these tumor and immune-related pathways, leading to a poor prognosis in CRC patients. POSTN, as a small extracellular matrix protein, plays a vital role in the regulation of cell-matrix interaction, which is considered to associate with TME and tumor progression [35]. Accumulated evidence suggested that POSTN promotes tumor metastasis by regulating immune responses [36, 37]. In our study, the GSEA results showed that high expression levels of POSTN were associated with many immune-related pathways, for instance, B cell receptor signaling pathway, chemokine signaling pathway, and cytokine-cytokine receptor interaction. It could be hypothesized that the regulation of immune-related signaling pathways may be involved in the regulatory role of POSTN in the clinical stage and prognosis in CRC.

Tumor-infiltrating immune cells (TIICs) are an important part of the complex TME that regulates tumorigenesis [38]. Therefore, we adopted the CIBERSORT algorithm in the present study to further explore the relationships of SPOCK1 and POSTN with the 22 TIICs subsets of immune reaction. The results showed that there were seven types of immune cells, plasma cells, CD4 memory resting T cells, monocytes, macrophages M0, macrophages M1, activated dendritic cells, and neutrophils, that had significantly different proportions based on the expression levels of SPOCK1. Similarly, the expression levels of POSTN were significantly correlated with infiltration levels of plasma cells, CD4 memory resting T cells, monocytes, macrophage M0, macrophage M2, and neutrophils. For validation, we used TIMER to analyze the correlation between the expression of SPOCK1 and POSTN with immune cell infiltration. In addition, we verified significant positive expressions of CD68 (macrophage M0) and CD206 (macrophage M2) in 8 cases of colorectal cancer using immunohistochemistry and immunofluorescence techniques. Our results showed that SPOCK1 and POSTN were significantly associated with immune cell infiltration. Altogether, these results indicated that SPOCK1 and POSTN had the strongest and consistent correlation with macrophage and neutrophil cells. It has been shown that increased intratumoral neutrophil in CRC is related to the acquisition of malignant phenotype and is an independent factor for the poor prognosis of CRC patients [39, 40]. The role of macrophages in CRC is controversial, but many studies have shown that tumor-infiltrating macrophages M2 promote the metastasis of CRC, leading to poor prognosis [41–44]. Zhang Y et al. found that SPOCK1 is related to the recruitment and differentiation of macrophages [45]. Recent evidence revealed that POSTN could recruit M2 tumor-related macrophages and promote malignant growth [46]. These are consistent with our conclusion. Taken together, it is possible that SPOCK1 and POSTN potentially regulate immune cell infiltration in the CRC microenvironment.

Immune checkpoint molecules are often associated with tumor cell immune evasion and tumor progression. In return, immune checkpoint inhibitors have shown great success in the treatment of CRC [25, 47]. Our results showed that SPOCK1 and POSTN expression were positively correlated with the expression of immune checkpoints, including PD-1, PD-L1, PD-L2, CTLA4, TIM-3, and B7-H3. PD-L1/2 suppresses T-cell function through the PD-1 receptor, causing tumor cells to escape from immune surveillance. Previous studies have shown that PD-1, PD-L1, PD-L2, and CTLA4 expressions were associated with a poor prognosis in CRC patients [48–51]. TIM-3 and B7-H3 are considered novel promising targets for immunotherapy. Recent researches have revealed that TIM-3 and B7-H3 are also involved in the evasion of cancer immune surveillance and CRC progression [52, 53]. These results suggest that SPOCK1 and POSTN may play a role in immune evasion, which partly explains their potential mechanisms for promoting tumor progression.

This study had some limitations. First, the roles of SPOCK1 and POSTN in CRC were analyzed based on TCGA or GEO data, so our results need to be verified with larger sample sizes. Second, the expression of SPOCK1 and POSTN was only verified in several cases of colon cancer due to limited resources. Therefore, subsequent experiments in vivo and in vitro should be required to confirm the concrete relationship between SPOCK1, POSTN, and infiltrating immune cells.

## Conclusion

In conclusion, SPOCK1 and POSTN were identified as key prognostic genes related to TME and were mainly expressed in CAF for CRC. High expression of SPOCK1 and POSTN was associated with the late clinic stage and predicted a poor prognosis. Further analysis indicated that the high levels of SPOK1 and POSTN were positively correlated with the M2 polarization level of macrophages, the infiltration level of neutrophils, and the expression level of immune checkpoints. Therefore, SPOCK1 and POSTN associated with CAF could be used as prognostic biomarkers and therapeutic targets in CRC.

## Supplementary Information

**Additional file 1**. **Fig. S1. **SPOCK1and POSTN mainly express in CAF for CRC. (**A**) Seven major clusters asepithelial, fibroblast, monocyte, endothelial, CMP, B, and T cells inGSE110009. (**B**) SPOCK1 and POSTN highly express in fibroblast cells inCRC. (**C**) Seven major clusters as epithelial, macrophage, fibroblast,tissue stem, endothelial, B, and T cellsin GSE120065. (**D**)SPOCK1 and POSTN highly express in fibroblast cells in CRC.

**Additional file 2**. **Fig. S2. **Forestmap shows the result of multivariate COX regression analysis for OSamong patients with CRC.

**Additional file 3**. **Fig.S3**Correlation of PD-1and TIM-3 with SPOCK1 and POSTN expressions in immunohistologicalstaining of CRCtissues. **(A, B) **SPOCK1expression is positively correlated with the expressions of PD-1 andTIM-3. **(C, D)**POSTN expression is positively correlated with the expressions ofPD-1 and TIM-3.Each dotrepresents a sample tissue.

**Additional file 4**. **TableS1**. Clinicopathological Characteristics of Colorectal Cancer Patients.

**Additional file 5**. **Table S2**. Patientcharacteristics of CRC from TCGA database.

## Data Availability

All the analysis data were accessed from the TCGA database (https://portal.gdc.cancer.gov/), GEO database (https://www.ncbi.nlm.nih.gov/geo/), and the KEGG pathway database ( www.kegg.jp/kegg/kegg1.html).
