# Supplementary material for: SPOCK1 and POSTN are valuable prognostic biomarkers and correlate with tumor immune infiltrates in colorectal cancer
Source: BMC Gastroenterol. 2023 Jan 7;23:4. doi: 10.1186/s12876-022-02621-2 (PMC9826581; doi:10.1186/s12876-022-02621-2)
Supplement: Supplementary file 1 — Additional file 1. Fig. S1. SPOCK1and POSTN mainly express in CAF for CRC. (A) Seven major clusters asepithelial, fibroblast, monocyte, endothelial, CMP, B, and T cells inGSE110009. (B) SPOCK1 and POSTN highly express in fibroblast cells inCRC. (C) Seven major clusters as epithelial, macrophage, fibroblast,tissue stem, endothelial, B, and T cellsin GSE120065. (D)SPOCK1 and POSTN highly express in fibroblast cells in CRC. [file 12876_2022_2621_MOESM1_ESM.docx]

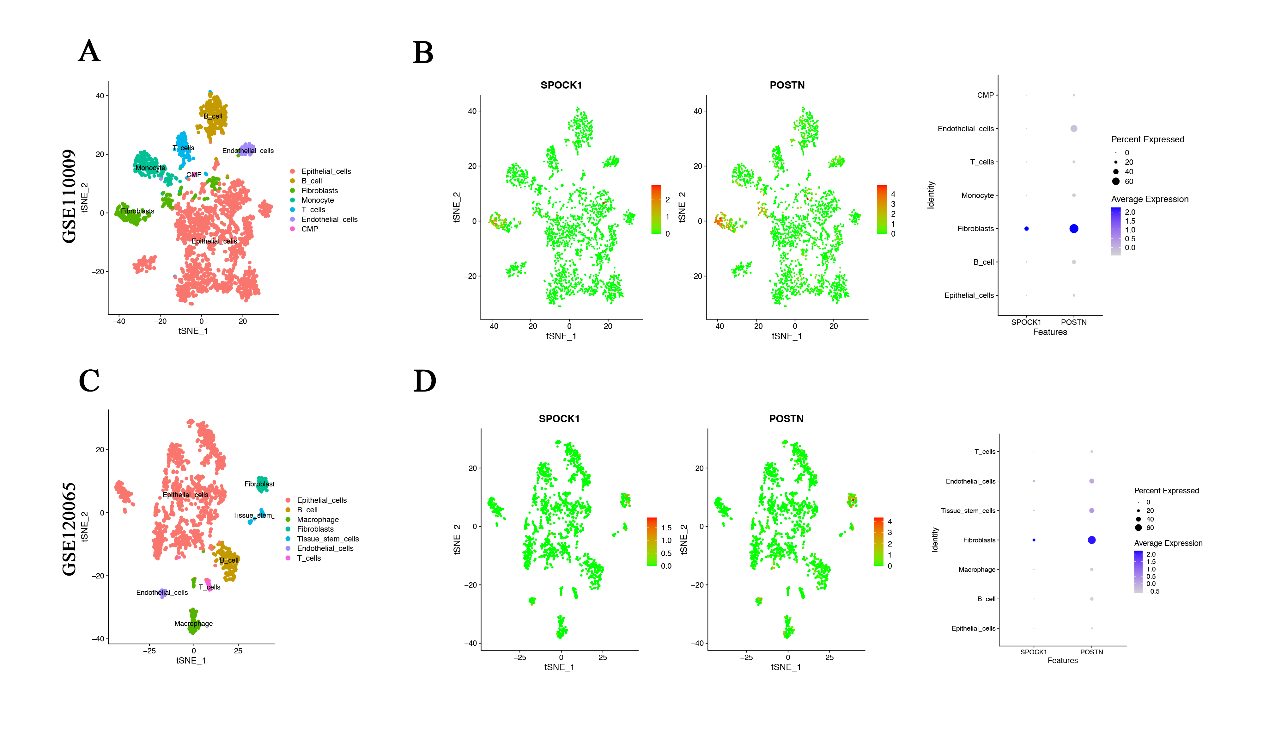


**Fig.S1** SPOCK1 and POSTN mainly express in CAF for CRC. (A) Seven major clusters as epithelial, fibroblast, monocyte, endothelial, CMP, B, and T cells in GSE110009. (B) SPOCK1 and POSTN highly express in fibroblast cells in CRC. (C) Seven major clusters as epithelial, macrophage, fibroblast, tissue stem, endothelial, B, and T cells in GSE120065. (D) SPOCK1 and POSTN highly express in fibroblast cells in CRC.
