# Supplementary material for: SPOCK1 and POSTN are valuable prognostic biomarkers and correlate with tumor immune infiltrates in colorectal cancer
Source: BMC Gastroenterol. 2023 Jan 7;23:4. doi: 10.1186/s12876-022-02621-2 (PMC9826581; doi:10.1186/s12876-022-02621-2)
Supplement: Supplementary file 2 — Additional file 2. Fig. S2. Forestmap shows the result of multivariate COX regression analysis for OSamong patients with CRC. [file 12876_2022_2621_MOESM2_ESM.docx]

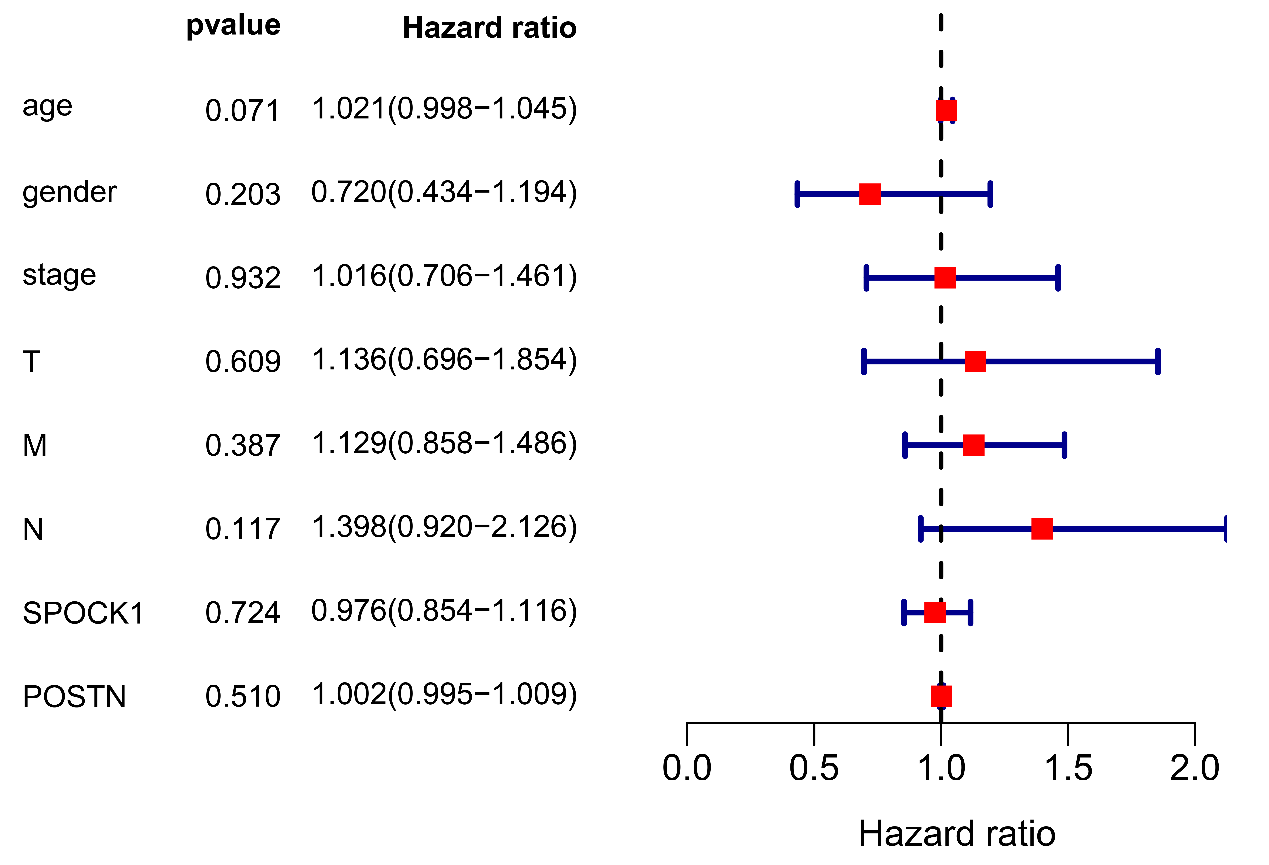


**Fig.S2** Forest map shows the result of multivariate COX regression analysis for OS among patients with CRC.
