# Supplementary material for: SPOCK1 and POSTN are valuable prognostic biomarkers and correlate with tumor immune infiltrates in colorectal cancer
Source: BMC Gastroenterol. 2023 Jan 7;23:4. doi: 10.1186/s12876-022-02621-2 (PMC9826581; doi:10.1186/s12876-022-02621-2)
Supplement: Supplementary file 3 — Additional file 3. Fig.S3Correlation of PD-1and TIM-3 with SPOCK1 and POSTN expressions in immunohistologicalstaining of CRCtissues. (A, B) SPOCK1expression is positively correlated with the expressions of PD-1 andTIM-3. (C, D)POSTN expression is positively correlated with the expressions ofPD-1 and TIM-3.Each dotrepresents a sample tissue. [file 12876_2022_2621_MOESM3_ESM.docx]

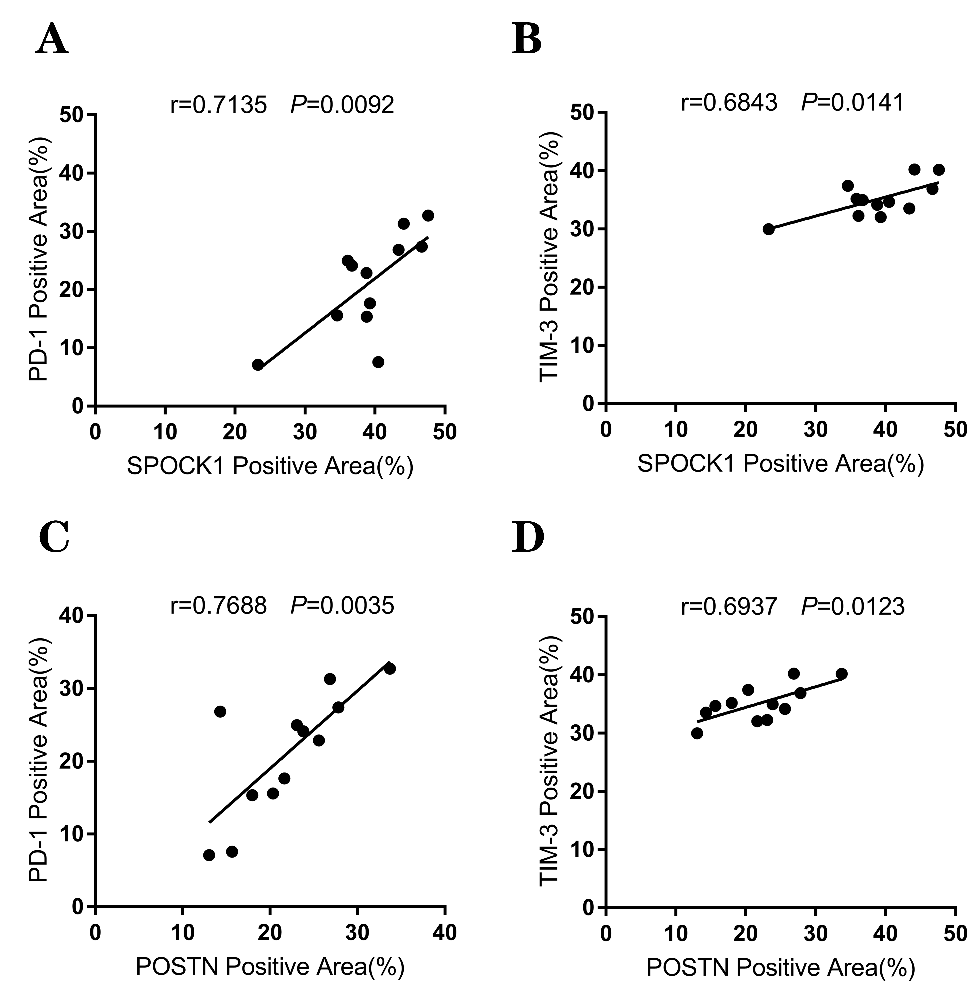


**Fig.S3** Correlation of PD-1 and TIM-3 with SPOCK1 and POSTN expressions in immunohistological staining of CRC tissues. **(A, B)** SPOCK1 expression is positively correlated with the expressions of PD-1 and TIM-3. **(C, D)** POSTN expression is positively correlated with the expressions of PD-1 and TIM-3. Each dot represents a sample tissue.
