# Supplementary material for: SPOCK1 and POSTN are valuable prognostic biomarkers and correlate with tumor immune infiltrates in colorectal cancer
Source: BMC Gastroenterol. 2023 Jan 7;23:4. doi: 10.1186/s12876-022-02621-2 (PMC9826581; doi:10.1186/s12876-022-02621-2)
Supplement: Supplementary file 4 — Additional file 4. TableS1. Clinicopathological Characteristics of Colorectal Cancer Patients. [file 12876_2022_2621_MOESM4_ESM.docx]

Table S1. Clinicopathological Characteristics of Colorectal Cancer Patients

| Patient Number | Gender | Age (years) | Smoking history | Histologic subtype | TNM | Histopathology | Date of surgery |
| --- | --- | --- | --- | --- | --- | --- | --- |
|  |  |  |  |  |  |  |  |
| 1 | Male | 68 | Yes | Adenocarcinoma | pT3N1Mx | G2(medium differentiation) | 10-Feb-21 |
| 2 | Male | 65 | No | Adenocarcinoma | pT3N0M0 | G2(medium differentiation) | 27-Aug-20 |
| 3 | Male | 66 | Yes | Adenocarcinoma | T4N0M0 | medium to low differentiation | 8-Jan-21 |
| 4 | Male | 49 | No | Adenocarcinoma | T4aN1M0 IIIB | G2(medium differentiation) | 4-Jan-21 |
| 5 | Female | 76 | No | Adenocarcinoma | pT4aN0M0 IIB | G2(medium differentiation) | 8-Jan-21 |
| 6 | Female | 62 | No | Adenocarcinoma | pT3N1M0 | G2(medium differentiation) | 8-Nov-21 |
| 7 | Female | 71 | Yes | Adenocarcinoma | pT4N2bM1 | G3(medium differentiation) | 8-Sep-20 |
| 8 | Female | 78 | No | Adenocarcinoma | pT3N2aM0 | G2(medium differentiation) | 29-Jan-21 |
