# Supplementary material for: SPOCK1 and POSTN are valuable prognostic biomarkers and correlate with tumor immune infiltrates in colorectal cancer
Source: BMC Gastroenterol. 2023 Jan 7;23:4. doi: 10.1186/s12876-022-02621-2 (PMC9826581; doi:10.1186/s12876-022-02621-2)
Supplement: Supplementary file 5 — Additional file 5. Table S2. Patientcharacteristics of CRC from TCGA database. [file 12876_2022_2621_MOESM5_ESM.docx]

Table S2. Patient characteristics of CRC from TCGA database.

| Clinical characteristics |  | | No.of patients  (511) | | % |
| --- | --- | --- | --- | --- | --- |
| Age (year)  Gender  Stage  T classification  M classification  N classification | young age (<60)  old age (≥60)  Male  Female  Ⅰ  Ⅱ  Ⅲ  Ⅳ  T1  T2  T3  T4  M0  M1  MX  N0  N1  N2 | 146  365  273  238  91  190  150  80  16  90  350  55  390  78  43  290  126  95 | | 28.6  71.4  53.4  46.6  17.8  37.2  29.3  15.7  3.1  17.6  68.5  10.8  76.3  15.3  8.4  56.7  24.7  18.6 | |
